# Pharmacokinetics of single- and multiple-dose flumatinib in patients with chronic phase chronic myeloid leukemia

**DOI:** 10.3389/fonc.2023.1101738

**Published:** 2023-02-06

**Authors:** Bo Jiang, Junyuan Qi, Mingyuan Sun, Weiwei Zheng, Yongyue Wei, Jianxiang Wang, Fengkui Zhang

**Affiliations:** ^1^ State Key Laboratory of Experimental Hematology, National Clinical Research Center for Blood Diseases, Institute of Hematology, Chinese Academy of Medical Sciences, Tianjin, China; ^2^ Department of Biostatistics, School of Public Health, Nanjing Medical University, Nanjing, China

**Keywords:** flumatinib, phase 3, pharmacokinetics, safety, chronic phase chronic myeloid leukemia

## Abstract

**Introduction:**

Flumatinib is a novel, oral breakpoint cluster region-abelson (BCR-ABL) tyrosine kinase inhibitor that has demonstrated manageable safety and promising efficacy in patients with newly diagnosed chronic phase (CP) chronic myeloid leukemia (CML).

**Methods:**

This study evaluated the pharmacokinetic (PK) profiles of flumatinib mesylate tablets at a dose of 400 mg and 600 mg in patients with CML-CP. The study was registered at chictr.org Identifier (ChiCTR2100044700). In this open-label, pharmacokinetic study, eligible patients were administered a single-dose of flumatinib 400 mg or 600 mg on day 1, followed by 2-day washout and 8 consecutive days of once-daily administration. Serial plasma samples were assayed for flumatinib and its metabolites (N-demethylate metabolite M1 and amide-bond hydrolytic metabolite M3).

**Results:**

Twenty-nine patients were assigned to flumatinib 400 mg (n=14) or 600 mg (n=15). Serum concentrations of flumatinib reached maximum measured plasma concentration (Cmax) at a median time of 2 hours after each single dose, and then eliminated slowly with a mean apparent terminal disposition half-life (t1/2) from 16.0 to 16.9 hours. Following single- and multiple-dose administration, flumatinib exposure (Cmax, area under the concentration-time curve from 0 to t hours (AUC_0-t_), area under the concentration-time curve from 0 hours to infinity (AUC_0-∞_)) increased in an approximately dose-proportional manner. There was approximately 4.1- and 3.4- fold drug accumulation at steady-state after multiple-dose administration at 400 mg and 600 mg, respectively. The drug-related AEs associated with both treatments were primarily low-grade and tolerable events.

**Conclusion:**

Analysis of PK parameters indicated that flumatinib exposure increased in an approximately dose-proportional manner. Further research needs to be conducted in a large sample-size study.

## Introduction

Chronic myeloid leukemia (CML) is a hematologic stem-derived malignancy characterized by the presence of a specific chromosomal translocation (chromosome 9, 22) known as the Philadelphia chromosome (Ph), leading to the constitutive activation of the breakpoint cluster region-abelson (BCR-ABL) tyrosine kinase, which plays a crucial part in the pathogenesis of CML (1, 2). Over the past decades, impressive therapeutic responses have been obtained with BCR-ABL tyrosine kinase inhibitors (TKIs) such as imatinib, nilotinib, dasatinb, bosutinib and radotinib in the first-line treatment of CML-chronic phase (CML-CP) (3, 4), making the life expectancy of patients with CML ultimately approaching that of the general population (5).

Studies over the past decades have demonstrated the predictive value of pharmacokinetic (PK) parameters using area under the curve (AUC) model or the threshold model for efficacy and safety metrics of the drugs (6). A subanalysis of the IRIS study demonstrated that imatinib trough levels were predictive of higher complete cytogenetic response (CCyR) independently of Sokal risk group (7). Population pharmacokinetic analysis of data from several dasatinib clinical studies showed that major cytogenetic response (MCyR) was significantly associated with weighted average steady-state plasma concentrations, and pleural effusion was significantly associated with trough concentration (8). Bosutinib exposure metrics estimated from a population pharmacokinetic model were observed to be identified with the pooled incidence (but not severity) of diarrhea and rash as well as CCyR, major molecular response (MMR), and cumulative complete hematologic response (CHR) at 1 year in patients with newly diagnosed CML-CP (9).

Flumatinib, with an optimized structure based on imatinib, is a potent, novel oral second-generation BCR-ABL TKI that has been put on the market in China. In the unpublished preclinical study, the peak concentration could be reached at an average of 5 h after oral administration of flumatinib with mean elimination half-life of 7.32 h in rats. Furthermore, flumatinib mesylate was widely distributed in the tissues of rats, mainly in the gastrointestinal tract, respiratory tract, liver, kidney and reproductive organs, in which the drug concentration was higher than that in plasma. The major drug-related components in circulation were the parent compound, *N*-desmethyl flumatinib (M1), a metabolite with similar pharmacological properties to flumatinib, and the amide hydrolytic metabolite (M3), which was approximately 30% of the parent drug in plasma (10). Besides, the combination of a high-fat diet with flumatinib would increase the bioavailability of flumatinib and M1 (11). In phase 1 (12) and unpublished phase 2 (in TKI treatment-Naïve patients with CML-CP) studies, it showed that the concentration of parent drug could reach steady state after 6-8 days of continuous administration and the molecular response was positively related with trough plasma concentration of flumatinib. Moreover, patients in 600 mg dosing group could reach higher rate of early molecular response than that of patients in 400 mg dosing group. Thus, we conducted the studies to verify the pharmacokinetic parameters of patients with CML-CP at doses of 400 mg and 600 mg and then to use the optimal dose in a larger sample-sized population. The FESTnd study published previously has demonstrated the efficacy and safety of Flumatinib 600 mg in newly-diagnosed patients with CML-CP (13). Here we reported the pharmacokinetic properties of flumatinib after single and multiple oral doses at a dose of 400 mg or 600 mg.

## Materials and methods

### Study design

This was an open-label study to evaluate the pharmacokinetics of flumatinib under fasted conditions after single and multiple consecutive doses in patients with CML-CP. According to the phase III (13) recommended dose, 12 subjects at least were scheduled to be enrolled in 400 mg group or 600 mg group respectively. Each patient received a single dose (400 mg or 600 mg) on day 1 followed by 2-day washout, and then on 4-11 days for 8 consecutive days of once-daily administration. The trial was conducted in accordance with the Declaration of Helsinki and the International Conference on Harmonization Guidelines for Good Clinical Practice. Written informed consent was obtained from all patients before they enrolled in the study. This trial has been approved by the Ethics Committee of National Clinical Research Center for Blood Diseases, Institute of Hematology, Chinese Academy of Medical Sciences. The data were collected with the use of electronic case report form (eCRF) and were analyzed by an outsourcing biostatistician. The study was registered at chictr.org Identifier (ChiCTR2100044700).

### Patients

Adult patients with CML-CP (18-65 years) with no documented T315I mutation, who were not expected to progress to accelerated phase (AP) or blast crisis (BC) within 3 months, who had not previously received tyrosine kinase inhibitors or any anti-leukemia treatments within 2 weeks (except hydroxyurea) were eligible in this study. Patients needed to have adequate organ function with an Eastern Cooperative Oncology Group (ECOG) performance status 0-1 (14). Patients without adequate heart, renal, hepatic, pancreatic, endocrine, metabolic, neurological, gastrointestinal, immunological, psychic or coagulation function, as well as normal electrolyte levels were prohibited. Patients with a history of neoplasm were not eligible. We also excluded patients under concomitant treatments that could inhibit or induce the activity of the liver enzyme cytochrome P450-3A4 or that may prolong the QT interval.

### Pharmacokinetic evaluations

For PK analysis of single-dose, blood samples were collected on day 1 at baseline 0 (pre-dose), 0.5, 1, 2, 3, 4, 6, 8, 10, 24, 48, and 72 h, respectively. In terms of PK analysis of multiple consecutive doses, patients were administered flumatinib 400 mg or 600 mg once daily for 8 consecutive days (D4-11) and blood samples were collected at baseline 0 (D11) and 0.5, 1, 2, 3, 4, 6, 8, 10, 24, 48, 72, 96, 120 and 144 h after the last dose (timeline table see **Supplemental Table S1**). For each plasma sample, 4 mL of blood was collected in a tube containing heparin, inverted and mixed 8 to 10 times, and centrifuged at 2000g for 10 min at 4°C after sample collection. The plasma was stored at - 70°C and transported to PK testing unit by cold chain. Plasma concentrations of flumatinib, and its metabolites (N-demethylate metabolite M1 and amide-bond hydrolytic metabolite M3) were determined by liquid chromatography tandem mass spectrometry (LC-MS/MS) (15). The serum concentration-time data of flumatinib mesylate were analyzed by a standard noncompartmental method using R software (version 4.0.2). The following parameters were calculated by PKNCA package: sampling time when maximum measured plasma concentration (T_max_) occurs, maximum measured plasma concentration(C_max_), minimum measured plasma concentration(C_min_), area under the concentration-time curve from 0 to t hours(AUC_0-t_), area under the concentration-time curve from 0 hours to infinity (AUC_0-∞_), apparent terminal disposition half-life(t_1/2_), Area under the first moment curve from 0 to t hours(AUMC_0-t_), Area under the first moment curve from 0 to infinity(AUMC_0-∞_), mean residue time (MRT_0-t_), total-body drug clearance for oral administration (CL/F), and volume of distribution based on the terminal phase (VZ/F).

The accumulation ratio Rac_Cmax_ and Rac_AUC_ were calculated as follows:


$${\text{Rac}}_{\text{Cmax}}={\text{C}}_{\text{max}}\left(\text{Day }11\right)/{\text{C}}_{\text{max}}\left(\text{Day }1\right)$$

$${\text{Rac}}_{\text{AUC}}={\text{AUC}}_{0-\text{t}}\left(\text{Day }11\right)/{\text{AUC}}_{0-\text{t}}\left(\text{Day }1\right).$$

### Safety evaluation

Safety was evaluated according to NCI CTC AE 4.03 including symptoms and signs, vital signs, blood routine, urine routine, blood biochemistry, coagulation function, electrocardiogram, ultrasound, chest X-ray, etc, until 28 days after the first dose.

### Statistical analysis

The sample size of 12 patients at least per flumatinib group met the National Medical Products Administration (NMPA) guideline for PK studies (16). The PK parameters of flumatinib mesylate were summarized by descriptive statistics, including arithmetic means, standard deviation, and coefficient of variation. Median values and ranges were provided for T_max_. Categorical data were analyzed by chi-square test or exact test, depending on which method best fit data. To analyze pharmacokinetic parameters between the two groups, an exploratory two-sample t-test was carried out using the log-transformed values of parameters. When comparing the parameters of single and multiple-dose administration, if the data were normally distributed, the paired two-sample t-test, or the signed rank sum test was adopted. Twenty-nine subjects were involved in the PK analysis of single dose. For PK analysis of multiple consecutive doses, the serum concentration-time data of patients numbered 209 in the 400 mg dose group and numbered 107 in the 600 mg dose group on day 11 of administration were missing and were not included in the analysis. Safety analyses included all patients who received more than 1 dose of Flumatinib. All the statistical analyses were conducted using R software (version 4.0.2).

## Results

### Patient characteristics

From 6 March 2017 to 3 August 2017, twenty-nine eligible patients with CML-CP were assigned to receive flumatinib 400 mg (n=15) or 600 mg (n =14). The cut-off date for this study was 31 August 2017, with 28 days of follow-up for the last enrolled patient. The demographic and baseline characteristics of all subjects involved in this study were presented in **Table 1**. Sex, age, ECOG performance status, Median hemoglobin, Median platelet count, and Median white-cell count were well-balanced in the two study groups.

**Table 1:** Demographic characteristics and baseline information of patients.

Characteristics	400 mg (n=15)	600 mg (n=14)	*P*
Median age (yr)	47.93 ± 11.45	46.36 ± 12.63	0.727
Sex, n			
Male	11	9	0.700
Female	4	5	
ECOG, n			
0	14	13	1.000
1	1	1	
Median hemoglobin (IQR)- g/L^#^	122 (145,67)	125.5 (149,83)	0.339
Median platelet count (IQR)-×10^9^/L	^#^	331 (1405,27)	354.5 (1081,101)	0.770
Median white-cell count (IQR)-×10^9^/L	^#^	5.29 (32.03,1.52)	12.53 (24.58,2.7)	0.192

^#^The description of hemoglobin, platelet and white cells was the median and analyzed by t test.

### Single-dose pharmacokinetics

**Figure 1:**
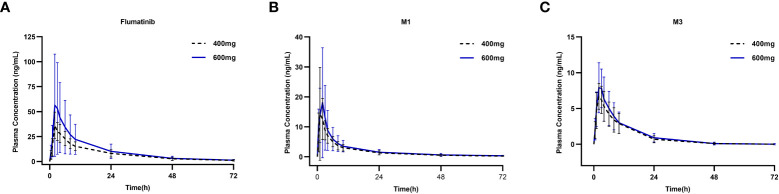
Mean plasma concentrations of flumatinib **(A)**, M1 **(B)** and M3 **(C)** on day 1 after oral administration of a single-dose of flumatinib 400 mg and 600 mg.

The mean plasma concentrations over time of flumatinib, M1, and M3 in patients with CML-CP on day 1 after oral administration for two dose levels (400 mg and 600 mg) are illustrated in **Figure 1**. The PK parameters of flumatinib, M1 and M3 derived from the plasma concentration-time curves on day 1 are summarized in **Table 2**. Flumatinib was rapidly absorbed with a median t_max_ of 2.0 hours and then eliminated slowly with a t_1/2_ from 16.0 to 16.9 hours following a single-dose of flumatinib 400 mg and 600 mg. Arithmetic mean C_max_ values of 38.0 ± 12.5 ng/ml and 61.9 ± 50.0 ng/ml, AUC_0–t_ values of 536.3 ± 179.9 ng·h/ml and 746.7 ± 534.7 ng·h/ml, and AUC_0–∞_ values of 564.3 ± 196.7 ng·h/ml and 777.4 ± 550.2 ng·h/ml were observed for 400 mg and 600 mg, respectively. A non-significant upward trend in C_max_, AUC_0–t_, or AUC_0–∞_ was observed between flumatinib 400 mg and 600 mg, suggesting that flumatinib exposure increased in an approximately dose-proportional manner.

**Table 2:** Pharmacokinetic parameters of flumatinib and two major metabolites following a single-dose administration.

Parameters	400 mg (n=15)	600 mg (n=14)	*P*
Flumatinib			
T_max_(h)^#^	2.0(2.0-6.0)	2.0(2.0-3.0)	0.3631
C_max_(ng/mL)	38.0 ± 12.5	61.9 ± 50.0	0.1242
AUC_0-t_ (h*ng/mL)	536.3 ± 179.9	746.7 ± 534.7	0.4185
AUC_0-∞_(h*ng/mL)	564.3 ± 196.7	777.4 ± 550.2	0.4230
CL/F(L/h)	793.0 ± 275.2	1254.9 ± 1314.2	0.2525
V_Z_/F(L)	19192.5 ± 7087.0	28670.1 ± 29618.0	0.3691
t_1/2_(h)	16.9 ± 2.4	16.0 ± 2.2	0.3181
AUMC_0-t_(h*h*ng/mL)	10102.0 ± 4396.7	12786.6 ± 8898.1	0.7114
AUMC_0-∞_(h*h*ng/mL)	12840.9 ± 6135.2	15709.2 ± 10420.3	0.6998
MRT_0-t_(h)	18.3 ± 2.1	16.9 ± 2.1	0.0977
M1			
T_max_(h)^#^	2.0(0.5-3.0)	1.5(0.5-3.0)	0.5334
C_max_(ng/mL)	17.3 ± 14.8	20.1 ± 17.8	0.4919
AUC_0-t_(h*ng/mL)	121.2 ± 49.1	150.6 ± 93.1	0.5681
AUC_0-∞_(h*ng/mL)	127.9 ± 52.2	160.8 ± 102.2	0.5489
CL/F(L/h)	3620.5 ± 1378	5603.3 ± 4824.6	0.1667
V_Z_/F(L)	96236.2 ± 47017	127464.3 ± 57764.9	0.0921
t_1/2_(h)	18.6 ± 5.1	18.9 ± 6.0	0.9662
AUMC_0-t_(h*h*ng/mL)	1817.3 ± 862.0	2296.3 ± 1440.5	0.7929
AUMC_0-∞_(h*h*ng/mL)	2485.9 ± 1203.2	3365.8 ± 2473.4	0.7009
MRT_0-t_(h)	14.7 ± 2.3	14.7 ± 3.8	0.7101
M3			
T_max_(h)^#^	2.0(1.0-4.0)	2.0(2.0-4.0)	0.1543
C_max_(ng/mL)	7.0 ± 1.9	8.8 ± 3.4	0.1358
AUC_0-t_(h*ng/mL)	68.4 ± 29.0	79.1 ± 34.4	0.5313
AUC_0-∞_(h*ng/mL)	74.3 ± 32.8	85.4 ± 34.9	0.4986
CL/F(L/h)	6621.0 ± 3642.5	9293.5 ± 7646.4	0.1505
V_Z_/F(L)	61964.3 ± 18494.8	92331.9 ± 33450.7	0.0053
t_1/2_(h)	7.4 ± 2.7	8.5 ± 3.4	0.5172
AUMC_0-t_(h*h*ng/mL)	660.1 ± 356.8	785.6 ± 497.8	0.7299
AUMC_0-∞_(h*h*ng/mL)	904 ± 517.1	1074.8 ± 623.2	0.6172
MRT_0-t_(h)	9.1 ± 2.5	9.1 ± 2.8	0.8641

^#^The T_max_ was analyzed by the median(maxium - minum) and the rest were described by the mean ± SD.

Similarly, the PK parameters of M1 and M3 showed a non-significant upward trend in C_max_, AUC_0–t_, and AUC_0–∞_ between two doses of Flumatinib.

### Multiple-dose pharmacokinetics

**Figure 2:**
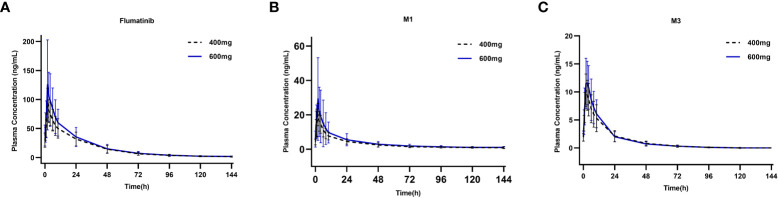
Mean plasma concentrations of flumatinib **(A)**, M1 **(B)** and M3 **(C)** at steady-state after oral administration of multiple-dose of flumatinib 400 mg and 600 mg.

The PK of flumatinib, M1, and M3 after multiple-dose administration at steady- state were also investigated. The mean plasma concentration of flumatinib, M1, and M3 analyzed on D11 are shown in **Figure 2**. The mean PK parameters at steady-state derived from the plasma concentration-time curves are summarized in **Table 3**.

**Table 3:** Pharmacokinetic parameters of flumatinib and two major metabolites following a multiple-dose administration.

Parameters	400 mg (n=15)	600 mg (n=14)	*P*
Flumatinib			
T_max_(h)^#^	2.0 (2.0-4.0)	2.0 (1.0-4.0)	0.5918
C_max_(ng/mL)	96.2 ± 25.0	129.0 ± 75.8	0.1613
C_min_(ng/mL)	29.5 ± 12.4	33.1 ± 14.3	0.4885
AUC_0-t_(h*ng/mL)	2211.9 ± 745.6	2570.4 ± 1060.9	0.3629
AUC_0-∞_(h*ng/mL)	2303.5 ± 779.0	2660.4 ± 1073.2	0.3785
CLss_F(L/h)	356.6 ± 93.2	458.0 ± 155.4	0.0793
V_Z__F(L)	18294.1 ± 8167.1	21774.6 ± 10791.5	0.4435
t_1/2_(h)	35.4 ± 12.1	32.3 ± 9.9	0.4957
AUMC_0-t_(h*h*ng/mL)	68519.4 ± 26904.3	75270.6 ± 31087.3	0.6423
AUMC_0-∞_(*h*ng/mL)	87421.1 ± 39295.5	92842.2 ± 35870.7	0.7120
MRT_0-t_(h)	30.6 ± 3.2	29.1 ± 3.3	0.3868
R_ac,AUC_	3.4	3.9	/
R_ac,Cmax_	2.9	2.6	/
M1			
T_max_(h)^#^	2.0 (1.0-4.0)	2.0 (0.5-3.0)	0.8881
C_max_(ng/mL)	21.0 ± 6.7	30.9 ± 23.5	0.1123
C_min_(ng/mL)	4.1 ± 1.7	5.2 ± 3.7	0.3730
AUC_0-t_(h*ng/mL)	415.5 ± 144.5	515.5 ± 312.9	0.2810
AUC_0-∞_(h*ng/mL)	498.5 ± 185.9	613.8 ± 333.7	0.2454
CLss_F(L/h)	2140.0 ± 833.9	2748.1 ± 932.8	0.1439
V_Z__F(L)	190521.8 ± 147843.6	282342.1 ± 204931.4	0.2130
t_1/2_(h)	64.8 ± 53.7	69.6 ± 44.0	0.6299
AUMC_0-t_(h*h*ng/mL)	15923.9 ± 6579.0	19040.2 ± 10041.8	0.3145
AUMC_0-∞_(h*h*ng/mL)	39482.6 ± 31880.1	45923.0 ± 29292.1	0.3202
MRT_0-t_(h)	37.7 ± 3.4	37.6 ± 3.8	0.7595
R_ac,AUC_	2.2	2.7	/
R_ac,Cmax_	1.6	1.9	/
M3			
T_max_(h)^#^	2.0 (1.0-4.0)	3.0 (2.0-4.0)	0.0595
C_max_(ng/mL)	10.6 ± 3.0	12.4 ± 4.3	0.2640
C_min_(ng/mL)	1.9 ± 0.7	1.8 ± 0.9	0.7163
AUC_0-t_(h*ng/mL)	166.2 ± 69.0	178.8 ± 67.1	0.5155
AUC_0-∞_(h*ng/mL)	172.4 ± 70.1	186.9 ± 67.7	0.4765
CLss_F(L/h)	3794.4 ± 1514.6	4881.3 ± 1546.8	0.0626
V_Z__F(L)	85639.2 ± 29325.5	125975.3 ± 54235.8	0.0396
t_1/2_(h)	16.4 ± 4.5	18.4 ± 7.6	0.5694
AUMC_0-t_(h*h*ng/mL)	3110.0 ± 1859.4	3030.5 ± 1477.4	0.8396
AUMC_0-∞_(h*h*ng/mL)	3754.2 ± 2113.4	3864.3 ± 1710.2	0.6699
MRT_0-t_(h)	17.5 ± 3.8	16.6 ± 3.7	0.8296
R_ac,AUC_	1.9	2.2	/
R_ac,Cmax_	1.5	1.6	/

^#^The T_max_ was analyzed by the median(maxium - minum) and the rest were described by the mean ± SD.

The median t_max_ after repeated administration of 400 mg or 600 mg was 2.0 hours, at which mean C_max_ were 96.2 ± 25.0 ng/mL and 129.0 ± 75.8 ng/mL with a non-significant upward trend. Similar trends were also seen in AUC_0–t_, and AUC_0–∞_. The mean plasma trough concentration of flumatinib at 400 mg and 600 mg at steady-state were 29.5 ± 12.4 and 33.1 ± 14.3 ng/mL respectively. The comparison of AUC_0-t_ and AUC_0-∞_ at steady-state and at day 1 demonstrated approximately 4.1- and 3.4- fold drug accumulation after oral administration at 400 mg and 600 mg, respectively. This accumulation had no significant difference between the two groups. Additionally, the t_1/2_ of flumatinib were 2.1- and 2.0-fold longer at steady-state than that of single-dose. Furthermore, MRT of flumaitnib increased nearly 2-fold at steady-state compared with Day 1 in both groups. Besides, the mean R_ac,AUC_ of flumatinib, M1, and M3 at 400 mg were 3.4, 2.2, and 1.9 while those at 600 mg were 3.9, 2.7, and 2.2, which suggested a cumulative effect, so as R_ac,Cmax_, though the differences had no statistical significance.

### Safety evaluations

Safety evaluations were performed on all patients (**Table 4**). Hematological adverse events (AE) especially neutropenia and thrombocytopenia occurred less frequently in 600 mg group (7.1%, 0%) compared with 400 mg group (13.3%, 13.3%). The most common non-hematological AE in 400 mg group was haematuria (26.7%) and diarrhea (13.3%) while diarrhea (28.6%), hypertriglyceridemia (21.4%), arthritis (21.4%), and nausea (14.3%) occurred more often in 600 mg group. Excluding one patient with arthritis of grade 3, which was not related to the treatment, all the non-hematological adverse events were of 1 to 2 grades. Generally, drug-related AEs associated with both treatments were primarily low-grade and tolerable events.

**Table 4:** AEs and newly occurring and worsen hematological or biochemical laboratory abnormalities.

AEs	400mg (n=15)			600mg (n=14)			Total (n=29)
	n(%)	Severe Grades		n(%)	Severe Grades		All Grades
		1-2 n(%)	3-4 n(%)		1-2 n(%)	3-4 n(%)	n(%)
Hematological Abnormalities							
Neutropenia	2(13.3%)	1(6.7%)	1(6.7%)	1(7.1%)	0	1(7.1%)	3(10.3%)
Thrombocytopenia	2(13.3%)	0	2(13.3%)	0	0	0	2(6.9%)
Anemia	0	0	0	1(7.1%)	1(7.1%)	0	1(3.4%)
Laboratory Abnormalities							
Hypertriglyceridemia	0	0	0	3(21.4%)	3(21.4%)	0	3(10.3%)
Lipase Elevation	1(6.7%)	1(6.7%)	0	1(7.1%)	1(7.1%)	0	2(6.9%)
Hyperbilirubinemia	0	0	0	1(7.1%)	1(7.1%)	0	1(3.4%)
ALT Elevation	0	0	0	1(7.1%)	1(7.1%)	0	1(3.4%)
AST Elevation	0	0	0	1(7.1%)	1(7.1%)	0	1(3.4%)
Hypocalcemia	1(6.7%)	1(6.7%)	0	0	0	0	1(3.4%)
Hyperuricemia	0	0	0	1(7.1%)	1(7.1%)	0	1(3.4%)
Non-hematological AEs							
Diarrhea	2(13.3%)	2(13.3%)	0	4(28.6%)	4(28.6%)	0	6(20.7%)
Hematouria	4(26.7%)	4(26.7%)	0	0	0	0	4(13.8%)
Arthritis	0	0	0	3(21.4%)	2(14.3%)	1(7.1%)	3(10.3%)
Dental Ulcer	1(6.7%)	1(6.7%)	0	1(7.1%)	1(7.1%)	0	2(6.9%)
Nausea	0	0	0	2(14.3%)	2(14.3%)	0	2(6.9%)
Rash	0	0	0	1(7.1%)	1(7.1%)	0	1(3.4%)
Angioedema	0	0	0	1(7.1%)	1(7.1%)	0	1(3.4%)
Alopecia	0	0	0	1(7.1%)	1(7.1%)	0	1(3.4%)
Fever	0	0	0	1(7.1%)	1(7.1%)	0	1(3.4%)
Abdominal Distension	0	0	0	1(7.1%)	1(7.1%)	0	1(3.4%)
Abdominal Pain	0	0	0	1(7.1%)	1(7.1%)	0	1(3.4%)
Pyspepsia	1(6.7%)	1(6.7%)	0	0	0	0	1(3.4%)
Phlebitis	0	0	0	1(7.1%)	1(7.1%)	0	1(3.4%)

## Discussion

Flumatinib is derived by replacing the phenyl ring of imatinib with a pyridine group and introducing trifluoromethyl group. These structural modifications allow flumatinib to achieve higher potency (17). Compared to other TKIs (imatinib, nilotinib, dasatinb, bosutinib and radotinib), the chemical structure of flumatinib is similar to imatinib, nilotinib, and radotinib. Flumatinib, like Nilotinib and radotinib, contains trifluoromethyl group (18).

In an unpublished phase 2 study, both flumatinib 400 mg and 600 mg exhibited significantly higher rate of MMR compared with imatinib in newly diagnosed CML-CP. Furthermore, flumatinib 600 mg was more effective than 400 mg, with a similar safety profile. The efficacy and safety of flumatinib 600mg was further confirmed in the subsequent phase 3 study (FESTnd). In the FESTnd study, compared with imatinib, flumatinib 600mg treatment demonstrated significantly higher rate of MMR, early molecular response (EMR), CCyR, and deep molecular response (DMR) in patients with CML-CP (13).

The present study was to demonstrate the pharmacokinetic profile of flumatinib at a dose of 400mg and 600mg. The t_max_ was the same between 400mg and 600mg after the single dose. A non-significant upward trend in C_max_, AUC_0–t_, or AUC_0–∞_ was observed between flumatinib 400 mg and 600 mg, suggesting that flumatinib exposure increased in an approximately dose-proportional manner after the single dose.

The PK profile of flumatinib after multiple-dose was compatible with once-daily dosing. After 6 days of consistent dosing once daily, the plasma concentration of flumatinib, M1, and M3 all reached the steady condition. The median t_max_ in the two groups was the same as each other, the maximum of which in the group 400mg was shorter than that of single-dose. Repeated dosing could increase the value of C_max_ and AUC and the Rac-_Cmax_ and Rac_-AUC_ were both more than 1 which was an implication of accumulation effect of flumatinib. The exposure of flumatinib of multiple-dose was 2-2.5 than that of single-dose. Moreover, C_min_ didn’t differ significantly between 400mg and 600mg and the varieties were close to each other. Likewise, the varieties between individuals of C_max_ were higher in the group of 600mg than 400mg. It was not clear whether the varieties in C_max_ had influenced the hematological responses. But the same C_min_ implicated that 600mg could be better to improve the exposure and could be cleared at the pace of 400mg. Eight patients in the group of 600mg and 6 patients in the group of 400mg had abnormal values of WBC at baseline. And most patients’ WBC could decrease to the normal range in one month after taking the first dose. One patient treated with 400mg and two patients treated with 600mg didn’t acquire hematological responses.

The PK properties of flumatinib are similar to those of imatinib in some respects. Both flumatinib and imatinib were absorbed rapidly after a single-dose administration with median t_max_ of 2.0 hours and 2–4 hours, respectively. The t_1/2_ of flumatinib in plasma was 16.0–16.9 hours, similar to that of imatinib (15 hours). Flumatinib exposure increased in an approximately dose-proportional manner. Similarly, the drug exposure of imatinib was dose proportional for the range of 25–1,000 mg (19). The PK properties of flumatinib differ slightly from those of nilotinib in some respects. After a single-dose administration, the absorption of nilotinib (median t_max_ occurring at 4 hours) was slightly slower than that of flumatinib. The t_1/2_ of nilotinib was greater (24.4 hours) than that of flumatinib. However, similar to flumatinib and imatinib, the drug exposure of nilotinib was nearly dose proportional for the range of 50–400 mg (20).

The safety profiles in the two groups were generally the same and most adverse events were tolerable. The hematological adverse events were more often in the group of 400mg than that in the group of 600mg mainly because more patients in the group of 400mg had normal peripheral blood cells at baseline. The most non-hematological adverse events were gastrointestinal abnormalities including omit, diarrhea, dental ulcer, abdominal discomfort, etc. Diarrhea occurred in six patients, 2 in group 400mg and 4 in group 600mg, and could take place more than once. Events such as rash, fever, alopecia, and angioedema appeared once in patients of group 600mg. The differences in occurrence rates of non-hematological events might result from more plasma exposure of 600mg. There is no denying that the toxicity evaluation just represented part of the safety profile during the early period after the first dose. More confirmed studies needed to be conducted to get more information about flumatinib in patients with CML-CP.

In conclusion, flumatinib exposure increased in an approximately dose-proportional manner. Since the difference was not statistically significant (flumatinib 600 mg vs. 400 mg), conclusions need to be drawn with caution. Further research needs to be conducted in a large sample-size study.

## Data availability statement

The raw data supporting the conclusions of this article will be made available by the authors, without undue reservation.

## Ethics statement

The studies involving human participants were reviewed and approved by Ethics Committee of National Clinical Research Center for Blood Diseases, Institute of Hematology, Chinese Academy of Medical Sciences. The patients/participants provided their written informed consent to participate in this study.

## Author contributions

BJ and JQ wrote the manuscript; JW and FZ designed the research; BJ, JQ, MS, and WZ performed the research; BJ and YW analyzed the data; YW contributed new reagents/analytical tools. All authors contributed to the article and approved the submitted version.
