# Supplementary material for: Pharmacokinetics of single- and multiple-dose flumatinib in patients with chronic phase chronic myeloid leukemia
Source: Front Oncol. 2023 Feb 6;13:1101738. doi: 10.3389/fonc.2023.1101738 (PMC9939828; doi:10.3389/fonc.2023.1101738)
Supplement: Supplementary file 1 [file Table_1.docx]

Supplementary Material

Table S1 | The PK sampling timeline.

|  | Time | Collection Time | Time window |
| --- | --- | --- | --- |
| Single-dose | D1 | 5min before the dose | ±5min |
|  |  | 0.5h after the dose | ±5min |
|  |  | 1h after the dose | ±5min |
|  |  | 2h after the dose | ±5min |
|  |  | 3h after the dose | ±5min |
|  |  | 4h after the administration | ±5min |
|  |  | 6h after the dose | ±5min |
|  |  | 8h after the dose | ±5min |
|  |  | 10h after the dose | ±5min |
| / | D2 | 24h after the dose | ±30min |
| / | D3 | 48h after the dose | ±30min |
| / | D4 | 72h after the dose | ±30min |
| Multiple doses | D4-D10 | / | / |
|  | D11 | 5min before the last dose | ±5min |
|  |  | 0.5h after the last dose | ±5min |
|  |  | 1h after the last dose | ±5min |
|  |  | 2h after the last dose | ±5min |
|  |  | 3h after the last dose | ±5min |
|  |  | 4h after the last dose | ±5min |
|  |  | 6h after the last dose | ±5min |
|  |  | 8h after the last dose | ±5min |
|  |  | 10h after the last dose | ±5min |
|  |  | 24h after the last dose | ±30min |
|  |  | 48h after the last dose | ±30min |
|  |  | 72h after the last dose | ±60min |
|  |  | 96h after the last dose | ±60min |
|  |  | 120h after the last dose | ±60min |
|  |  | 144h after the last dose | ±60min |
